# Wilson disease in pregnancy: A case series

**DOI:** 10.1097/MD.0000000000032968

**Published:** 2023-02-17

**Authors:** Xiali Xiong, Hong Wei, Yunxia Zhu, Xin Zhou, Zhiqiang Zhao, Qiang Chen

**Affiliations:** a Department of Obstetrics, Beijing You’An Hospital, Capital Medical University, Beijing, China; b China Academy of Chinese Medical Sciences Eye Hospital, Beijing, China.

**Keywords:** coagulopathy, liver cirrhosis, outcomes, pregnancy, Wilson disease

## Abstract

Hepatolenticular degeneration, also known as Wilson disease (WD), is an autosomal recessive inherited disease characterized by copper metabolism, which has complex clinical manifestations, and mainly including liver and nervous system lesions. Pregnancy combined with WD is extremely harmful to mothers and children, with high miscarriage rates, and premature birth rates and perinatal mortality.

**Patient concerns:**

Here we introduced the basic information of 4 pregnant women with WD. The first pregnant woman had a 16-year history of WD, stopped taking penicillamine 1 year before pregnancy. The second woman had a 3-year history of WD and was taking penicillamine regularly, unintended pregnancy occurred 1 month after stopping the drug. The third woman had a history of WD for 5 years with penicillamine treatment. The 4th woman was found to have WD due to repeated missed miscarriage with abnormal liver function, after which penicillamine was regularly taken. Fortunately, she was pregnant again a year later.

**Diagnoses:**

The pregnant women in case 1 and case 2 were diagnosed with decompensated cirrhosis with coagulation dysfunction during pregnancy. The pregnant woman in case 3 was found to have liver cirrhosis by ultrasound, and the pregnant woman in case 4 did not have liver abnormalities during pregnancy.

**Interventions:**

The pregnant woman in case 1 began to take copper-removing drugs and take a low-copper diet after finding the aggravation of the disease in the early stage of pregnancy, and had good compliance during pregnancy. The pregnant woman in case 2 had poor compliance during pregnancy and did not receive any treatment. The pregnant woman in case 3 refused to use copper elimination drugs during pregnancy, but took a low copper diet. The pregnant woman in case 4 had good compliance during pregnancy, and she was treated with drugs and low copper diet during the whole pregnancy.

**Outcomes:**

Three of the four pregnant women got a healthy baby but premature, and only the pregnant woman in case 2 had spontaneous abortion at 25 weeks.

**Lessons:**

After comprehensive monitoring and multidisciplinary management of professional medical staff before and after pregnancy, WD pregnant women still have the opportunity to obtain a better pregnancy outcome and improve quality of life.

## 1. Introduction

Hepatolenticular degeneration, also known as Wilson disease (WD), is a copper metabolism disorder caused by mutations in the gene ATPase copper transporting beta located on chromosome 13q14. The abnormal metabolism of copper in the body leads to excessive deposition of copper in the liver, brain, kidney, cornea and other tissues, and causing corresponding organ damage. The clinical manifestations of the disease are complex, there may be no clinical symptoms, or acute hepatitis, acute liver failure, chronic hepatitis, cirrhosis and other liver manifestations, as well as dystonia, tremor, limb stiffness and motor retardation, and mental behavioral abnormalities and other neurological symptoms. Additionally, it can also show Kayser-Fleischer ring in the eyes, as well as abnormalities in the kidney, heart, bone and joint system, blood system, and reproductive system and endocrine system.^[1–3]^ For WD women of childbearing age, in addition to common liver and nervous system diseases, menstruation reduction or amenorrhea, difficulty in pregnancy, and repeated miscarriages are also common.^[4]^ Due to the application of copper displacement therapy such as penicillamine and zinc, patients can often obtain similar reproductive rights as normal people. However, some women with WD experience worsening of liver disease or neuropsychiatric symptoms during pregnancy, progressing to decompensated cirrhosis or explosive liver failure.^[5]^ The incidence rate of this disease is low, with a worldwide prevalence of 1/2600 to 1/30,000, and fewer reports of WD during pregnancy.^[6]^

Therefore, this article reported the pregnancy outcomes of 4 pregnant women with WD admitted to our hospital in the past 20 years and discussed the pregnancy management of WD pregnant women. Each patient has obtained written informed consent before medication. This study has been approved by the ethics committee of Beijing You’An Hospital affiliated to Capital Medical University.

## 2. Case presentations

### 2.1. Case 1

A 36-year-old woman with a history of WD for 16 years, who had 2 pregnancies and 1 child, 1 preterm birth vaginally at 33 weeks gestation and 1 spontaneous miscarriage at 20 weeks gestation. She stopped taking penicillamine half a year before pregnancy. She came to our hospital at 12 weeks of pregnancy and was advised to terminate pregnancy due to decompensated coagulation of liver cirrhosis, but the pregnant woman and her family firmly refused. She had regular pregnancy tests during pregnancy and began to take penicillamine again at 12 weeks’ gestation, along with low-copper diet. She had intermittent bleeding in the gums and nasal mucosa. At the 29th week of pregnancy, due to obvious abnormality of serum indicators, and she was admitted to hospital for symptomatic treatment. At the time of admission, the serum ceruloplasmin was 0.121 g/L (the normal value was 0.2–0.6 g/L). At the 32nd week of pregnancy, she was terminated by cesarean section under general anesthesia due to the aggravation of her condition. During the operation, ascites was about 1000 mL, and the amount of bleeding was 1000 mL. The woman was transferred to Intensive Care Unit for further treatment. The premature infant developed mild asphyxia and was transferred to the neonatal intensive care unit. The newborn and maternal condition was stable at 1 month follow-up.

### 2.2. Case 2

A 23-year-old woman with a history of WD for 3 years, diagnosed with compensated cirrhosis 2 years ago, and regularly took penicillamine. She had not been pregnant before, 1 month after stopping the medication she found out that she was pregnant. No pregnancy tests were done during pregnancy. She complained of bleeding from gums and nasal mucosa during pregnancy, which had not been seen before. At 22 weeks of pregnancy, she was diagnosed decompensated cirrhosis with coagulation dysfunction, but she refused the suggestion of treatment. When she came to our hospital, she was 25 weeks pregnant with contractions, anemia, edema (++++), and skin scattered in old ecchymoses. There are many abnormalities in her serum indicators, including ceruloplasmin (0.090 g/L). Two days after admission, she delivered a dead baby, with about 700 mL bleeding during delivery. She was transferred to the medical ward for further treatment after delivery. After a month of follow-up, her physical condition was good.

### 2.3. Case 3

A 22-year-old woman with a history of WD for 5 years, and regularly took penicillamine. She was not pregnant before. She stopped medication after pregnancy and had irregular tests during pregnancy. At 33 weeks of gestation, a healthy infant was born prematurely and then transferred to neonatal intensive care unit. The maternity admission examination was normal, except for ceruloplasmin (0.113 g/L). At the same time, ultrasonic examination showed cirrhosis. She continued to be treated with penicillamine for copper-excretion after delivery. The condition of neonate and parturient was good after 1 month follow-up.

### 2.4. Case 4

A 26-year-old woman had 2 missed abortions during early pregnancy, the third missed abortion was treated in our hospital and diagnosed with WD, and with the serum ceruloplasmin 0.078 g/L. After that, penicillamine was taken regularly to eliminate copper. She conceived again after 1 year of treatment, continued penicillamine treatment during pregnancy, delivered vaginally at 36 weeks of pregnancy, and with ceruloplasmin 0.145 g/L before delivery. The premature infant had no deformity and grew well.

## 3. Discussion

WD disease can lead to infertility and habitual abortion, so the cases of WD combined with pregnancy are rare. The exact reason is still unclear. On 1 hand, it may be due to the weakening of the inactivation of estrogen after copper deposition in the liver through the negative feedback of the hypothalamus-pituitary-ovarian axis. As a result, the secretion of gonadotropins was reduced, leading to ovarian ovulation dysfunction and ultimately manifested as menstrual disorders and infertility. On the other hand, the ‘natural copper ring ’formed by excessive copper deposition in the endometrium plays a contraceptive role. It greatly increases the risk of miscarriage and intrauterine death by affecting the implantation of fertilized eggs.^[3,4,7]^ In recent years, WD patients have gradually recovered their ovulation function and improved their fertility after systematic copper excretion treatment. At present, cases of WD patients with pregnancy and successful pregnancy have been reported 1 after another. In our case, the 1st, 3rd, and 4th pregnant women had successful pregnancy after treatment, and the outcome was satisfactory. These examples suggest that WD pregnant women have the hope of being a mother, may have better pregnancy outcomes after standardized treatment and guidance in professional hospitals.

### 3.1. Treatment of WD during pregnancy

The treatment principle of WD is early treatment, individualized treatment, lifelong treatment, and lifelong monitoring. Patients with early diagnosis and early treatment have a good prognosis, but discontinuation of treatment is likely to lead to deterioration.^[8,9]^ Therefore, in addition to the guidance of low-copper diet after pregnancy, WD women of childbearing age need to continue to take drugs for copper-excretion treatment. Drug treatment of WD during pregnancy is more important. The role of the drug is to promote excretion of copper (copperchelating agents such as D-penicillamine and trichostatin) and reduce absorption of copper (such as zinc).^[4,10,11]^ There are relatively few pregnant women with WD, and the side effects of drugs on mothers and children remain to be tracked. European association for the study of the liver and American association for the study of liver diseases guidelines recommend that treatment doses of chelators (including D-penicillamine and trichostatin) should be reduced during pregnancy and that clinical symptoms and liver function, as well as blood copper and urinary ketone, should be regularly monitored during pregnancy, and while postdelivery doses should be increased to prepregnancy levels.^[10–12]^ In our report, the second and third pregnant women had poor compliance during pregnancy and did not take drugs to continue copper removal treatment. In case 2, the disease deteriorated during pregnancy, and the liver cirrhosis progressed from compensatory period to decompensatory period, eventually leading to adverse pregnancy outcomes. Although the pregnant woman in case 3 obtained a surviving fetus after interrupting treatment during pregnancy, her condition had progressed to liver cirrhosis. However, the pregnant woman in case 1, despite having a severe condition, and was able to carry the pregnancy to term and deliver a surviving fetus through consistent adherence to a low-copper diet and copper exclusion treatment. Case 4 pregnant woman demonstrated good compliance during pregnancy, leading to a successful conception. These results indicated that drug treatment of WD during pregnancy can reduce the disease and help to obtain better pregnancy outcomes.

### 3.2. Treatment of WD during delivery

The method of termination of pregnancy in WD pregnant women should be based on the patient complications and obstetric conditions. When WD is stable and organ involvement is mild, vaginal trial production can be selected when there is a chance of vaginal trial production; when WD pregnant women have indications for obstetric surgery or complicated with decompensated cirrhosis, coagulation dysfunction, gastric varices, and it is recommended to terminate pregnancy by cesarean section. However, careful operation is needed during the operation, active hemostatic treatment is also needed. In order to avoid the occurrence of abdominal wall hematoma, drainage tubes can be placed on the abdominal wall as needed when selecting the lower abdominal transverse incision. In the case of coagulation dysfunction, the incidence of postpartum hemorrhage will be significantly increased. We must be vigilant for potential bleeding issues during and after the surgery, take active measures to prevent and treat infection. It is necessary to actively correct coagulation dysfunction and hypoproteinemia after operation, it is better to carry out dynamic monitoring of liver function.^[4,8]^ In this paper, 2 pregnant women had decompensated cirrhosis and coagulation dysfunction. One pregnant woman terminated pregnancy by cesarean section, with intraoperative bleeding of 1000 mL. Another pregnant woman had spontaneous abortion, with bleeding of 700 mL. Both had a good prognosis after active rescue.

### 3.3. Prenatal diagnosis of WD

WD is an autosomal recessive genetic disease. WD patients can marry and give birth normally after treatment with stable symptoms, but genetic counseling is recommended.^[13,14]^ Prenatal genetic diagnosis is recommended if the spouse is a carrier or a couple who has given birth to a child with WD is pregnant again. This can allow individuals with WD to make informed decisions about whether to continue the pregnancy. Prenatal diagnosis methods include villi biopsy, amniocentesis, umbilical cord blood puncture, or mutation detection of ATPase copper transporting beta gene in fetal cells. Noninvasive prenatal testing is also used for prenatal diagnosis of WD.^[15–17]^

## 4. Conclusion

Pregnancy in women with WD can be challenging due to the high risk of miscarriage, premature birth, and perinatal mortality. However, with careful monitoring and management by medical professionals, WD pregnant women can still achieve successful pregnancies and improve their quality of life. It is important to monitor liver function and provide treatment with copper-excretion medications during pregnancy in order to mitigate the risk of complications. Prenatal genetic testing can also be helpful in determining the likelihood of a successful pregnancy in couples where 1 partner has WD or where a previous child has been diagnosed with the disease.

## Author contributions

**Conceptualization:** Xiali Xiong, Qiang Chen, Yunxia Zhu.

**Data curation:** Xiali Xiong, Wei Hong, Xin Zhou, Zhiqiang Zhao.

**Validation:** Xin Zhou, Xiali Xiong, Qiang Chen.

**Writing – original draft:** Xiali Xiong, Qiang Chen.

**Writing – review & editing:** Qiang Chen.
